# Percutaneous Coronary Intervention versus Coronary Artery Bypass Grafting for Chronic Total Occlusion of Coronary Arteries: A Systematic Review and Meta-Analysis

**DOI:** 10.1155/2023/9928347

**Published:** 2023-11-06

**Authors:** Chenyang Wang, Sheng Liu, Raimov Kamronbek, Siyao Ni, Yunjiu Cheng, Huiyuan Yan, Ming Zhang

**Affiliations:** ^1^Center for Coronary Heart Disease, Beijing Anzhen Hospital, Capital Medical University, Beijing, China; ^2^Key Laboratory on Assisted Circulation, Ministry of Health, Department of Cardiology, The First Affiliated Hospital, Sun Yat-Sen University, Guangzhou, China; ^3^Department of Cardiology, Hangjinqi People's Hospital, Hangjinqi, Mongolia

## Abstract

**Introduction:**

Chronic total occlusion (CTO) of coronary arteries constitutes a substantial clinical challenge and has historically been managed through medical management and coronary artery bypass grafting (CABG). However, with the advancement in interventional technology, the success rate of percutaneous treatment has been significantly improved, and percutaneous coronary intervention (PCI) has emerged as a primary mode of treatment for CTOs, demonstrating remarkable clinical efficacy. The objective of this systematic review and meta-analysis is to evaluate and contrast the outcomes of PCI and CABG in patients with CTO.

**Methods and Results:**

A systematic search was conducted in the databases of PubMed, Embase, and Web of Science. The primary endpoints evaluated in this meta-analysis were the occurrence of major adverse cardiac events (MACE) and all-cause mortality. Secondary endpoints included myocardial infarction (MI), cardiac death, and the need for repeat revascularization. Nine studies, encompassing a total of 8,674 patients, were found to meet the criteria for inclusion and had a mean follow-up duration of 4.3 years. The results of the meta-analysis revealed that compared to CABG, PCI was associated with a lower incidence of all-cause mortality (RR: 0.78, 95% CI: 0.66–0.92; *P* = 0.003) and cardiac death (RR: 0.55; 95% CI: 0.31–0.96; *P* < 0.05), but an increased risk of myocardial infarction (MI) (RR: 1.96; 95%CI: 1.07–3.62; *P* < 0.05) and repeat revascularization (RR: 7.13; 95% CI: 5.69–8.94; *P* < 0.00001). There was no statistically significant difference in MACE (RR: 1.11; 95% CI: 0.69–1.81; *P* = 0.66) between the PCI and CABG groups.

**Conclusion:**

In the present meta-analysis comparing PCI and CABG in patients with chronic total occlusion of the coronary arteries, the results indicated that PCI was superior to CABG in reducing all-cause mortality and cardiac death but inferior in decreasing myocardial infarction and repeat revascularization. There was no statistically significant difference in MACE between the two groups.

## 1. Introduction

Chronic total occlusion of the coronary artery refers to a pathological condition characterized by complete occlusion of the coronary artery, a TIMI blood flow grade of 0, and persistence for more than three months [1]. The prevalence of chronic total occlusion (CTO) is substantial, with a reported incidence of 15% to 20% of coronary heart disease patients undergoing coronary angiography examination in multiple large-scale, multicenter studies [2, 3]. A majority of these patients receive optimal guideline-directed medical therapy (GDMT), while others are treated through revascularization, and a minority undergo percutaneous coronary intervention (PCI) [4]. PCI for CTO lesions presents more challenges and complications than non-CTO lesions, with relatively lower success rates. Nevertheless, the success rate of CTO-PCI in experienced centers has improved significantly to 80% to 90% due to advancements in equipment, technology, and practitioner expertise [5, 6]. A multicenter, randomized controlled trial, involving a cohort of 396 patients, has demonstrated that CTO-PCI can relieve anginal symptoms and enhance quality of life when contrasted with the administration of optimal medical therapy (OMT) alone [7]. Compared to failed CTO-PCI, successful CTO-PCI is associated with the lower incidence rates of mortality, stroke, repeat revascularization, and recurrent angina [8]. In addition, a prospective study that included 1,777 patients demonstrated that CTO-PCI significantly improves survival and reduces the 1-year incidence of MACCE [3]. Presently, the principal benefit associated with CTO-PCI is regarded as the improvement of symptoms, while research data on whether it affects patients' long-term prognosis remains limited.

PCI and coronary artery bypass grafting (CABG) are two modalities for revascularization of patients with CTO. However, the impact of these two methods of revascularization on the prognosis of patients with CTO is still controversial. Hence, this meta-analysis aims to provide a more comprehensive understanding by comparing the PCI and CABG treatment strategies for CTO patients, incorporating all available cohort studies.

## 2. Methods

### 2.1. Search Strategy

A systematic review was conducted by searching PubMed, Embase, and Web of Science databases for studies that compared PCI and CABG in patients with CTO. The search criteria utilized both MeSH terms and text words including “chronic total occlusion,” “percutaneous coronary intervention,” “coronary artery bypass grafting,” and “revascularization.” The search was performed from the year 2000 to March 2023, with no language restrictions, and included both fully published research and abstracts. This meta-analysis was registered with the International Prospective Register of Systematic Reviews (PROSPERO ID: CRD42022326498).

### 2.2. Inclusion and Exclusion Criteria

The studies included in this meta-analysis compared the outcomes between PCI and CABG in patients with CTO. The outcomes evaluated included all-cause mortality, MI, cardiac death, repeat revascularization, and the incidence of MACE.

The following studies were excluded from this meta-analysis: (1) studies comparing the outcomes between successful and unsuccessful PCI in patients with CTO; (2) studies comparing the outcomes between PCI and CABG in CTO patients who also had other illnesses; (3) studies that exclusively focused on one treatment strategy; and (4) studies conducted on animal subjects.

### 2.3. Study Selection

**Figure 1:**
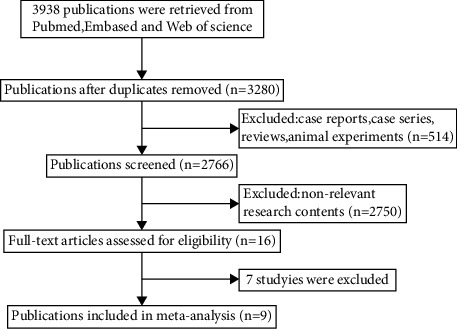
Flow diagram of the studies selection process.

Our initial search generated 3,938 references (Figure 1). Of these, 3,922 (99.6%) were excluded from title and summary searches due to duplication, irrelevant content, animal subjects, unreported results of interest, or other reasons. The remaining 16 studies were reviewed in full, and 7 were excluded because they did not report results of interest. Finally, nine studies [3, 9–16] met the inclusion criteria.

### 2.4. Data Extraction

The process of data extraction was performed by two researchers, W.C.Y. and L.S., using a standardized form. Any discrepancies were resolved through discussion between the researchers. The following information was collected: the author's name, year of publication, location of study participants, study design, age and gender of participants, and relevant results.

### 2.5. Outcomes

The primary focus of this meta-analysis was to assess the incidence of MACE and all-cause mortality. MACE was defined as a composite of cardiac death, cerebrovascular accident, MI, or repeat revascularization. The secondary outcomes were MI, cardiac death, and repeat revascularization.

### 2.6. Methodological Quality

The process of study selection, data collection and analysis, and reporting of results adhered to the guidelines set forth by the Epidemiological Observational Study (MOOSE) group [17]. We use the Newcastle-Ottawa Scale to appraise the quality of the studies (Table 1).

Weighted risk ratios (RRs) and 95% confidence intervals (CIs) were calculated for categorical variables. We used Cochrane Q-statistic and *I*^2^-statistic to perform heterogeneity analysis [18]. We used a fixed-effects model to combine effect sizes when heterogeneity was insignificant (*I*^2^ < 50%). When heterogeneity was significant (*I*^2^ ≥ 50%), we used a random-effects model. We conducted sensitivity analysis by eliminating each study in turn to assess the effect of each study on the pooling RRs. Funnel plots were performed to assess publication bias. We used RevMan 5.3 software (The Cochrane Collaboration, The Nordic Cochrane Centre, Copenhagen, Denmark) to perform our analyses.

## 3. Results

### 3.1. Characteristics of Studies

As shown in Table 2, the base characteristics of the nine studies that we included were displayed. Of these, one is a randomized controlled trial (RCT), three are prospective cohort studies, and five are retrospective cohort studies. Our meta-analysis included 8674 patients, of whom 4466 underwent PCI and 4208 underwent CABG. The mean duration of follow-up was 4.3 years, and the internal validity of the eligible studies was evaluated as moderate, as depicted in Table 1.

### 3.2. All-Cause Mortality

**Figure 2:**
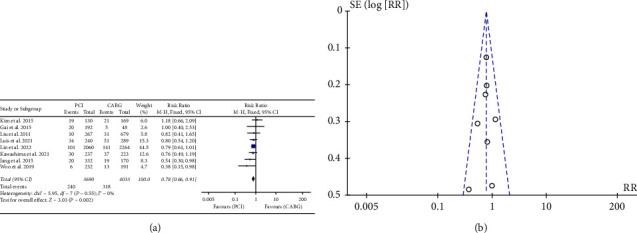
(a) Forrest plot for all-cause mortality. CABG = coronary artery bypass grafting; PCI = percutaneous coronary intervention; and CI = confidence interval. (b). Funnel plot for all-cause mortality.

The meta-analysis of 7723 patients revealed that 558 individuals, constituting 7.0% of the total sample, passed away during the follow-up period. The results indicated that PCI was correlated with a reduced incidence of all-cause mortality in comparison to CABG (RR: 0.78, 95% CI: 0.66–0.92; *P* = 0.003). (Figure 2(a)).

### 3.3. MACE

**Figure 3:**
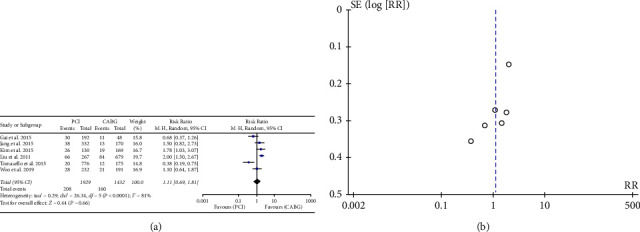
(a) Forrest plot for MACE. CABG = coronary artery bypass grafting; PCI = percutaneous coronary intervention; CI = confidence interval; MACE = major acute cardiovascular event. (b). Funnel plot for MACE. MACE = major acute cardiovascular event.

Of the nine studies examined, six reported a total of 368 MACE over the course of the follow-up period. The results indicated that PCI did not result in a lower incidence of MACE compared to CABG (RR: 1.11, 95% CI: 0.69–1.81; *P* = 0.66). (Figure 3(a)).

### 3.4. Myocardial Infarction

**Figure 4:**
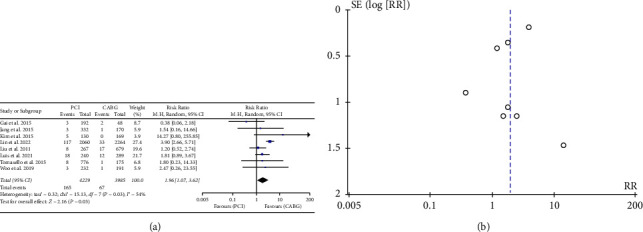
(a) Forrest plot for MI. CABG = coronary artery bypass grafting; PCI = percutaneous coronary intervention; CI = confidence interval; MI = myocardial infarction. (b). Funnel plot for MI. MI = myocardial infarction.

Of the nine studies analyzed, eight reported 232 instances of MI during the follow-up period. The results indicated that the incidence of MI was higher in the group undergoing PCI compared to the group receiving CABG, and the difference was found to be statistically significant (RR: 1.96, 95% CI: 1.07–3.62; *P* < 0.03). (Figure 4(a)).

### 3.5. Cardiac Death

**Figure 5:**
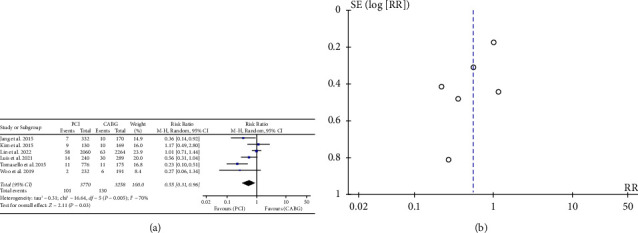
(a) Forrest plot for cardiac death. CABG = coronary artery bypass grafting; PCI = percutaneous coronary intervention; CI = confidence interval. (b). Funnel plot for cardiac death.

Of the nine studies evaluated, six reported 231 cases of cardiac deaths during the follow-up period. The results indicated that the incidence of cardiac death was lower among individuals receiving PCI compared to those who underwent CABG (RR: 0.55; 95% CI: 0.31–0.96; *P* = 0.03). (Figure 5(a)).

### 3.6. Repeat Revascularization

**Figure 6:**
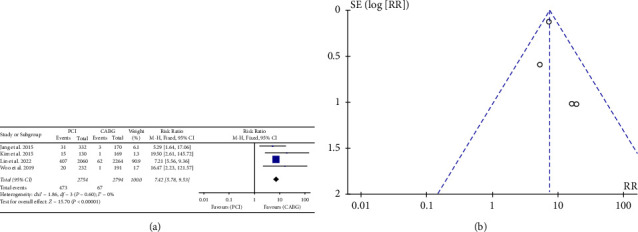
(a) Forrest plot for repeat revascularization. CABG = coronary artery bypass grafting; PCI = percutaneous coronary intervention; CI = confidence interval. (b). Funnel plot for repeat revascularization.

Four of the nine studies in question revealed that there were 540 cases of repeat revascularization documented during the follow-up period. The result revealed that the incidence of repeat revascularization was higher among individuals who underwent PCI as compared to those who received CABG (RR: 7.42, 95% CI: 5.78–9.53; *P* < 0.00001). (Figure 6(a)).

### 3.7. Publication Bias Analysis

In our meta-analytic study, we utilized funnel plots to examine the presence of publication bias among all the studies that were included (Figures 2(b)–6(b)).

### 3.8. Sensitivity Analysis

**Figure 7:**
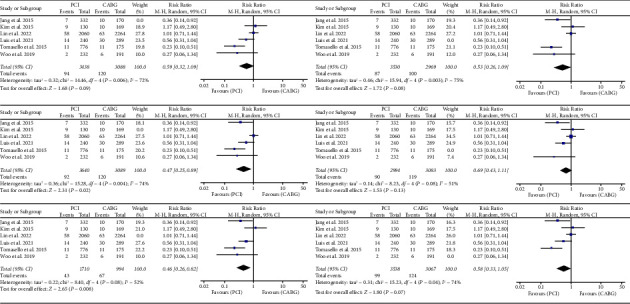
Results of sensitivity analysis for cardiac death. CABG = coronary artery bypass grafting; PCI = percutaneous coronary intervention; CI = confidence interval.

**Figure 8:**
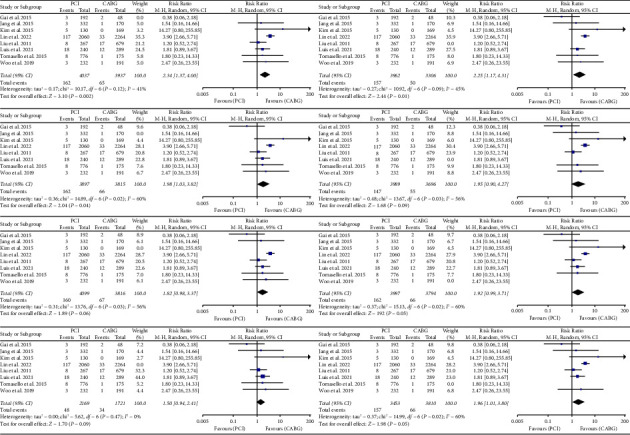
Results of sensitivity analysis for MI. MI = myocardial infarction.

**Figure 9:**
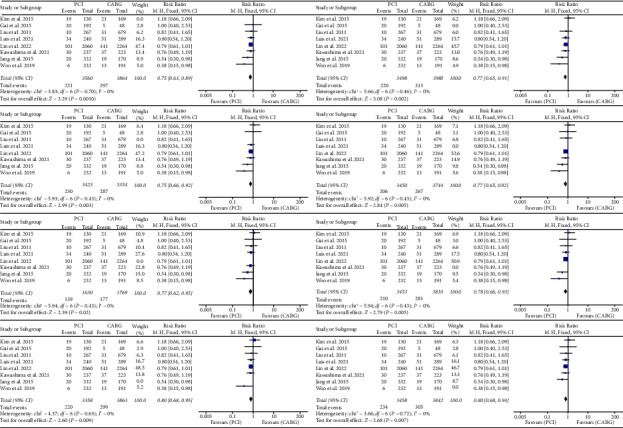
Results of sensitivity analysis for all-cause mortality.

We performed a leave-one-out meta-analysis to assess the influence of each individual study on the combined Relative Risks (RRs). The results of these sensitivity analyses showed that there was no noticeable alteration in the combined RRs, suggesting that the results are robust and stable (Figures 7–9).

## 4. Discussion

To date, our study constitutes the first meta-analytic examination that focuses on comparing the clinical outcomes of PCI and CABG in the context of CTO patients. Our findings indicate that, compared to CABG, PCI has lower all-cause mortality and the occurrence of cardiac death, but a higher incidence of myocardial infarction and repeat revascularization. However, there were no statistically significant differences observed between the PCI and CABG groups in terms of the incidence of MACE.

CTO affects a range of 15–20% of patients with CAD [19]. Nonetheless, only a small proportion of CTO patients, ranging from 5 to 14%, undergo PCI [2, 20]. The management of patients with CTO is impacted by various factors, including the number of obstructed vessels, prior myocardial infarction status, and the presence of angina pectoris [21]. The advancement in technology and equipment has expanded the indications for CTO-PCI and improved its outcomes [22]. Several studies advocate revascularization as the optimal treatment strategy for CTO patients [9, 11, 15]. Despite ongoing debates regarding the optimal revascularization strategy, our meta-analysis has provided evidence that PCI is superior to CABG in terms of reducing all-cause mortality and the occurrence of cardiac death. This outcome could potentially be attributed to the advancements in interventional techniques in recent years.

Advancements in coronary guidewire design, operating techniques, and the utilization of the J-CTO score can aid in optimizing the choice of revascularization strategies to maximize benefits [23–25]. In particular, the use of vascular imaging techniques such as intravascular ultrasound (IVUS) in PCI has substantially enhanced clinical outcomes. In a comprehensive meta-analysis conducted by Jang et al. that encompassing a cohort of 24,849 patients, IVUS-guided PCI significantly reduced the incidence of MACE events, all-cause mortality, MI, target vessel revascularization, and stent thrombosis in patients compared with angiography-guided PCI [26]. For CTO-PCI, the influence of IVUS on procedural outcomes remains controversial. The results of a randomized controlled trial led by Kim et al. demonstrated that IVUS-guided CTO-PCI significantly reduced the 12-month incidence of MACE in patients compared with the angiography group (hazard ratio: 0.35, 95% CI: 0.13–0.97) [27]. Another randomized controlled trial showed that there was no significant difference in the 2-year rates of MACE between the IVUS-guided and angiography-guided groups. Nevertheless, it is worth noting that within the IVUS-guided group, there were marked reductions in the rates of late in-stent lumen loss and stent restenosis [28]. The meta-analysis by Panuccio et al. included 2 randomized controlled trials and 3 observational studies encompassing 2,320 patients, which demonstrated that the IVUS-guided group significantly reduced the incidence of in-stent thrombosis compared with the angiography-guided group [29]. The application of IVUS in CTO-PCI specifically pertains to the optimization of stent-related parameters during CTO interventions, such as stent length and diameter and the assessment of the proximal cap and calcifications [30].

It is noteworthy that CTO-PCI is typically performed on low-risk patients with single-vessel disease, while high-risk patients with multi-vessel coronary disease, left main involvement, and a decreased left ventricular ejection fraction are more likely to undergo CABG [3, 9, 14]. This could also contribute to the lower all-cause mortality and occurrence of cardiac death observed in the PCI group. The widespread use of second-generation drug-eluting stents is also thought to contribute to the improved outcomes of PCI [16].

However, our meta-analysis indicates that PCI is inferior in reducing myocardial infarction compared to CABG, and no significant difference in MACE was observed between the two revascularization methods. This finding is aligned with the results of the BEST and SYNTAX trials [31, 32], which showed that although the rate of myocardial infarction was higher in the PCI group than in the CABG group, it did not result in increased mortality. In addition, our meta-analysis also confirms the previously reported higher incidence of repeat revascularization in the PCI group compared to the CABG group. This may be due to the use of the internal mammary artery (IMA) grafts in the CABG group, which has been associated with improved long-term patency rates [33].

In medical practice, the selection of a revascularization strategy typically involves consideration of several factors such as the extent of coronary artery disease, the physical state of the patient, and the patient's preferences. Our study's findings indicate that CABG does not possess a clear superiority in treating CTO. Conversely, our results demonstrate that PCI is more effective than CABG in reducing the all-cause mortality and the incidence of cardiac death, which could assist physicians in making more informed revascularization decisions for CTO patients. To our knowledge, our study is the first meta-analysis focusing on the effect of PCI and CABG on the prognosis of patients with CTO. Although there is a meta-analysis examining the effects of three treatment methods (OMT, PCI, and CABG) on CTO patients [34], it mainly focuses on revascularization and OMT, which is quite different from our study. Moreover, we included more studies, which changed the impact of these two types of revascularization on the prognosis of patients with CTO. In addition, the end points of our study were more comprehensive.

## 5. Study Limitations

The choice of revascularization strategy for CTO patients by physicians may be influenced by various factors, including ACEF (age, creatinine, and ejection fraction), SYNTAX I, and SYNTAX II scores. However, our meta-analysis did not consider these factors as subgroups in the choice of management strategy, as the necessary data was not available in the original literature. In addition, the nine studies included in our meta-analysis had varying follow-up times, clinical outcomes, and criteria for enrollment, contributing to increased heterogeneity and affecting the generalizability of the results. The limited number of randomized controlled trials in our meta-analysis, as well as non-random factors such as patient characteristics and patient and physician preferences, may also impact the results. The number of CTO vessels may also affect clinical outcomes, but our meta-analysis did not address this aspect as relevant data was lacking.

## 6. Conclusion

In the present meta-analysis comparing PCI and CABG in patients with chronic total occlusion of the coronary arteries, the results indicated that PCI was superior to CABG in reducing all-cause mortality and cardiac death, but inferior in decreasing myocardial infarction and repeat revascularization. The meta-analysis did not reveal a significant difference in MACE between the two groups. Further investigation is necessary to establish the most effective approach for revascularization in cases of CTO.

## Figures and Tables

**Table 1:** Quality of studies as per Newcastle Ottawa scale.

Serial numbers	Study	Study design	Selection	Comparability	Outcome
1	Gai et al.	Retrospective	★★★	★	★★★
2	Kawashima et al.	Randomized	★★★	★	★★★
3	Lin et al.	Prospective	★★★★	★	★★★
4	Liu et al.	Prospective	★★★	★	★★★
5	Kim et al.	Retrospective	★★★★	★	★★
6	Woo et al.	Retrospective	★★★	★	★★★
7	Tomasello et al.	Prospective	★★★	★	★★
8	Woo et al.	Retrospective	★★★	★	★★★
9	Luis et al.	Prospective	★★★	★	★★

**Table 2:** Characteristics of studies.

First author	Publication year	Region	Sample size (n)	Age (mean ± SD)	Male (%)	Follow-up
Gai et al.	2015	China	253	—	—	5 years

Kawashima et al.	2021	Netherlands	480	PCI: 64.7 ± 10.3	PCI: 79.3	10 years
				CABG: 64.5 ± 10.5	CABG: 85.2	

Lin et al.	2022	China	4324	PCI: 57.5 ± 10.6	PCI: 83.7	5 years
				CABG: 60.9 ± 9.1	CABG: 83.0	

Liu et al.	2011	China	6000	PCI: 60.16 ± 10.53	PCI: 74.5	3 years
				CABG: 61.47 ± 9.71	CABG: 83.2	

Kim et al.	2015	Republic of Korea	2024	CABG: 61.1 ± 9.6	CABG: 87.0	46.5 months
				PCI: 62.0 ± 11.1	PCI: 86.9	

Woo et al.	2019	Republic of Korea	2019	CABG: 62.9 ± 9.9	CABG: 83.8	32 months
				PCI: 63.1 ± 11.1	PCI: 80.2	

Tomasello et al.	2015	Italy	1777	CABG: 68.8 ± 8.9	CABG: 84	12 months
				PCI: 67.0 ± 10.6	PCI: 84.8	

Woo et al.	2015	Republic of Korea	738	—	—	3.5 years

Luis et al.	2021	Spain	1248	PCI: 62.8 ± 10.8	PCI: 85	4.3 years
				CABG: 65.3 ± 9.5	CABG: 87	

CABG = coronary artery bypass grafting; MACE = major adverse cardiac event; MI = myocardial infarction; PCI = percutaneous coronary intervention.
